# Dermatology in black skin^⋆^

**DOI:** 10.1016/j.abd.2023.10.001

**Published:** 2024-02-02

**Authors:** Maurício Mota de Avelar Alchorne, Katleen da Cruz Conceição, Leonardo Lora Barraza, Marilda Aparecida Milanez Morgado de Abreu

**Affiliations:** a Faculty of Medicine, Universidade Nove de Julho, São Paulo, SP, Brazil; b Department of Dermatology, Santa Casa de Misericórdia do Rio de Janeiro, Rio de Janeiro, RJ, Brazil; c Department of Dermatology, Universidade do Oeste Paulista, Presidente Prudente, SP, Brazil

**Keywords:** Dermatopathies, Ethnicity, Pigmentation, Population, Skin

## Abstract

The vast majority of publications in dermatology refer to lightly pigmented skin, with few addressing the peculiarities of black skin. In addition there is no consensus on what it means to be black in different regions of the world. The lack of knowledge on the subject makes it difficult to recognize and manage dermatoses in this type of skin. This article aims to review the literature on intrinsic characteristics, as well as epidemiological and clinical aspects of the cutaneous manifestations of different dermatoses in black skin. It was found that there are sometimes striking differences, in the structural, biological, and functional aspects when comparing lightly pigmented and black skin. There are also physiological changes that need to be recognized to avoid unnecessary interventions. Some dermatoses have a higher incidence in black skin, such as acne, eczema, dyschromia and dermatophytosis. On the other hand, several dermatoses are more specific to black skin, such as pseudofolliculitis barbae, keloid, dermatosis papulosa nigra, ulcers caused by sickle-cell anemia, dactylolysis spontanea, confluent and reticulated papillomatosis of Gougerot and Carteaud, and some diseases of the hair and scalp (including fragile and brittle hair, traction alopecia, folliculitis keloidalis nuchae, folliculitis dissecans and central centrifugal cicatricial alopecia). A spectrum of peculiar aspects of specific dermatoses, including sarcoidosis, lichen planus (with emphasis on the pigmentosus variant), psoriasis, lupus erythematosus, vitiligo, syphilis, pityriasis versicolor, and neoplasms are highlighted. In the latter, characteristics of basal cell carcinoma, squamous cell carcinoma, and melanoma are compared, in addition to highlighting unusual aspects of primary cutaneous T-cell lymphoma, endemic Kaposi sarcoma, and dermatofibrosarcoma protuberans.

## Dermatology in black skin

The vast majority of dermatology publications refer to light skin, with few addressing the peculiarities of black skin. This lack of knowledge on the subject makes it difficult to recognize dermatoses on black skin and can generate fear among dermatologists about carrying out more invasive procedures on these patients, especially regarding the induction of scars and pigmentary changes.^1^, ^2^

## The black population

There is no single definition of what it means to be black. Therefore, this subjectivity must be taken into account when interpreting studies. According to anthropologists, there are five races: Caucasoids, Mongoloids, Australoids, Negroids/Congoids and Capoids). Ethnicity refers to a social group with linguistic, cultural and ancestral affinities.^1^, ^2^, ^3^, ^4^ In the Brazilian Portuguese dictionary, the definition of black is “one with a very dark color; of black skin". Taking into account Fitzpatrick's classification of phototypes, black people fall into types IV, V and VI, those who rarely or never burn in the sun and tan easily.^1^, ^3^

While in the United States, black is someone who has a black ancestor at any point in the family tree, regardless of skin tone, in Brazil, the Brazilian Institute of Geography and Statistics (IBGE, *Instituto Brasileiro de Geografia e Estatística*) differentiates people by skin color, which can be white, black, yellow, brown and indigenous, considering brown and black people as black.^3^ The term “*pardo*” (brown) applies to the miscegenation between Indians, whites and blacks, that is, people with indigenous, European and African ancestry.^3^ Due to the high degree of miscegenation of the Brazilian population, there is little precision in identifying a black person, with the criterion of self-declaration prevailing for statistical purposes.^3^

According to data from the National Household Sample Survey (PNAD Contínua, *Pesquisa Nacional por Amostra de Domicílios*) 2021, 43% of the Brazilians declared themselves as white, 47% as mixed race and 9.1% as black.^4^

## Structural, biological and functional aspects of black skin

### Epidermis

#### Stratum corneum

There is no significant variation in the stratum corneum thickness, although black people have a greater number of layers which are more compact and cohesive.^5^, ^6^ In lightly pigmented skin, 6 to 15 strips of tape are needed to remove the stratum corneum; in black skin, from 8 to 25. Due to greater cohesion, vesicles and bullae remain intact longer than in lightly pigmented skin.^7^

#### Lipid composition

Black skin presents lower levels of ceramides and greater transepidermal water loss,^5^, ^7^ in addition to a 2.5-fold higher rate of spontaneous desquamation than white skin, justifying greater xerosis in this population.^8^

#### Melanocytes/melanosomes

There is no difference in the number of melanocytes in black skin, but rather in the characteristics of the melanosomes, which are larger, non-aggregated and degrade more slowly compared to those in white skin. Its distribution also differs, present throughout the epidermis. Additionally, melanosomes have a more neutral pH and greater tyrosinase activity in black skin.^5^, ^7^, ^9^, ^10^

There is greater melanogenesis in black people and the melanin content naturally confers a Sun Protection Factor (SPF) of 13.4 compared to 3.4 for white individuals.^11^ Dark skin transmits 7.4% of ultraviolet rays (UVR) B compared to 29.4% in lightly pigmented skin,^7^, ^12^, ^13^ that is, approximately four times less UV reaches the upper dermis, minimizing photoaging and the propensity for skin tumors.^7^, ^12^ However, as UVR deteriorates the defense system, black skin also requires photoprotection.^7^, ^12^

The relative composition of eumelanin and pheomelanin is independent of the degree of pigmentation, but there is a low level of photoprotective eumelanin in lightly pigmented skin, explaining the greater sensitivity to UVR. ^14^

### Dermis

There is no difference regarding the thickness of the dermis between ethnic groups, although there is a difference at the cellular level.^5^, ^7^, ^9^ The collagen fiber bundles are smaller, macrophages are larger and more numerous, whereas mast cells differ only in the size of the granules, which are larger in black skin.^5^, ^7^, ^9^ Fibroblasts are larger, more numerous, bi- or multinucleated and hyperreactive, and are, associated with a constitutional decrease in collagenase, explaining the greater predisposition to keloids.^1^ These characteristics protect against the effects of age, delaying dermal atrophy in black people. There are no differences regarding elastic tissue.^7^, ^9^

### Adnexal glands

Studies on differences in adnexal glands present conflicting results. Some report that there are no differences in the number of eccrine glands. However, most agree that apocrine glands are more numerous and larger in black skin and also produce a larger amount of secretion, with a characteristic odor.^5^, ^7^ The sebaceous glands do not differ in number, but they are larger and produce a larger amount of sebum,^7^ which can favor the appearance of acne lesions.^8^

### Scalp, hair follicles and hair

#### Scalp

The scalp presents macules that resemble asterisks, scales and cosmetic residues, with erythema being common. The color, seen in trichoscopy, varies between light brown and black and does not correlate with the skin color, with a perifollicular pigmented network, or honeycomb pattern, consisting of pigmented lines (corresponding to the melanocytes of the crest of the network) surrounding hypochromic areas (fewer melanocytes in the suprapapillary epidermis). A unique and normal characteristic is the presence of white dots between the follicular units, similar to the fibrosis sign of cicatricial alopecia.^15^

#### Hair follicles

The density and number of hair follicles are lower and there are differences in their shape: they are curved, spiral-shaped, elliptical on cross-section. There is an asymmetry in the shape and cellular distribution of the hair bulb and the internal and external root sheaths.^16^, ^17^ The melanosomes are present in the external root sheath and in the bulb of vellus hairs, which is why they are more heavily pigmented. The sebaceous glands are fewer and less active, secreting less sebum, leading to the dryness.^16^, ^17^, ^18^ Blood flow is reduced, increasing susceptibility to cicatricial alopecia. The elastic tissue anchorage to the hair follicle is reduced, favoring traction alopecia.^7^

#### Hair

Negroid hair is the most distinct phenotypic characteristic of black people. They are difficult to comb, requiring different care and less frequent washing. Hairs present knots, cracks, and longitudinal fissures along the shaft.^7^, ^19^, ^20^ There are varying degrees of curl, with mechanical fragility increasing with higher degrees of curvature. The shaft has a smaller diameter, thinner cuticle, and is drier (lower water and sebum content), with lower tensile strength, which favors breakage.^1^ Several extracellular matrix proteins and adherens junctions are decreased,^16^, ^17^ leading to differences in hair texture, strength, and manageability. The follicular units commonly have hair shafts emerging together and the wavy fibers emerge at an angle to the scalp. The chemical composition of keratin and amino acids is similar to that of hair of other ethnic groups. Small differences have been reported in the number of low- and high-sulfur proteins (disulfide bonds).^17^, ^18^ When placed in water, African-American hair has a lower percentage of radial absorption than Asian and Caucasian hair, perhaps due to differences in lipid content.^16^, ^19^, ^20^

The main differences between black skin and lightly pigmrnted skin are shown in Table 1.

**Table 1:** Differences between black skin and lightly pigmented skin.

Characteristics	Black skin	Lightly pigmented skin
Stratum corneum	Same thickness	Same thickness
	Compact stratum corneum cells, with great intercellular cohesion. More cell layers	Less compact cells
		Fewer cell layers
Melanocytes	Equal number	Equal number
Melanosomes	Elongated, not grouped, present throughout the epidermis	Small, grouped, absent in the superficial layers of the epidermis
Melanin content	Higher	Smaller
Hair	Spiral	Smooth, helical, wavy
Follicular degeneration syndrome	++	–
Traction alopecia	++	–
Mast cells	Large granules	Small granules
Pruritus	++	+
Collagen fibers	Small and stacked	Fiber fragments occasionally present.
Scarring and keloid formation	+++	+
Fibroblasts	Long and in greater number	Small and fewer in number
Vitamin D production	+	+++

Modified from Zaid, 2017.^7^

## Dermatoses in black skin

**Figure 1:**
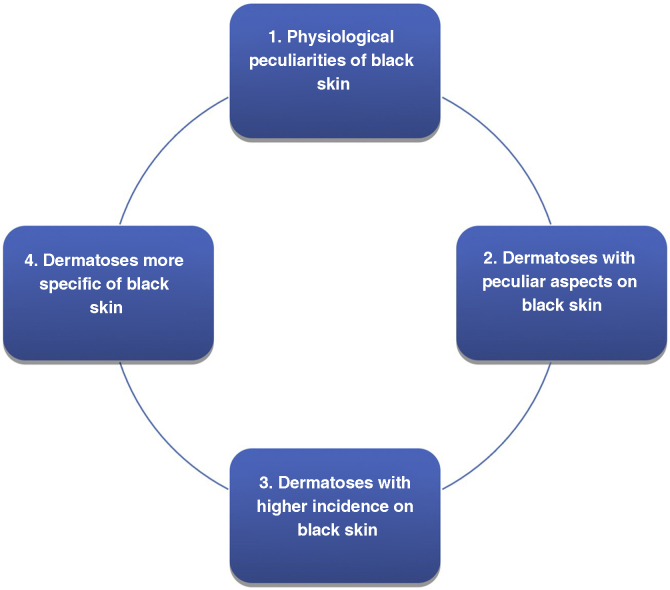
Highlighting topics in black skin.

There are variations in black skin that can be mistaken for abnormalities. On the other hand, some dermatoses are more frequent or practically exclusive to black people, while others present in a particular way, with characteristics inherent to the higher degree of pigmentation and a tendency to peculiar reaction patterns. These particularities require training for their identification and appropriate handling and are classified in Fig. 1 in several topics.^1^, ^3^

Black people tend to present four pathological reaction patterns:^1^ (1) Pigmentary lability (tendency to form pigmentary lesions, resulting in hyperpigmentation and hypopigmentation); (2) Follicular response and follicular diseases; (3) Mesenchymal response (fibroblastic and granulomatous); (4) Bullous response.

It is worth mentioning that red or brown dermatoses on lightly pigmentated skin, appear black, gray or purple on black skin because of the heavy pigmentation. Follicular, papular, and annular lesions are more common in Afro-Caribbean individuals.^7^

## Physiological peculiarities of black skin

The physiological changes of black skin are shown in Table 2.^21^, ^22^, ^23^, ^24^, ^25^, ^26^, ^27^

**Table 2:** Physiological peculiarities of black skin.

Alteration	Incidence	Etiopathogenesis	Clinical characteristics
Melanosis of the malar area^21^	Equal regarding gender; older than 50 years	Unknown. In young women, it seems to be related to atopy	Hyperchromia, with imprecise borders, symmetrically distributed in the malar regions
Perioral hyperpigmentation^21^	Higher in females	Unknown. It does not seem to be a consequence of inflammation	Hyperchromia in the skin adjacent to the angles of the mouth
Periorbital melanosis^22^	Higher in the female sex	Multifactorial: genetics, post-inflammatory hyperpigmentation, periorbital edema, hypervascularization and shadowing	Bilateral periorbital and eyelid hyperchromia, sometimes compromising the upper part of the nose and glabella. There is an increase in the amount of melanin in the papillary dermis, melanophages and vasodilation in the reticular dermis
Maturational hyperpigmentation^22^	4‒5th decades of life. Fitzpatrick skin phototypes IV‒V	Chronic sun exposure and possibly obesity and diabetes	Hyperchromic patches, with ill-defined edges that eventually disappear into normal skin, affecting areas exposed to the sun, including the sides of the face and the back of the hands and feet. On histopathology, proliferation of melanocytes with reports of papillomatous epidermal proliferation
Voigt-Futcher lines^7^, ^23^	17% to 40% of blacks; higher in women	Unknown. Congenital or acquired (hormonal -pregnancy)	Well-defined bilateral lines, which separate more pigmented areas from lighter areas. It can occur on the limbs, chest or abdomen, along the dermatomes
Midline hypopigmentation^7^	30% to 40% of blacks; higher in males; less visible with age	Autosomal dominant inheritance	Hypochromic linear band on the sternal region, which may extend to the linea alba and infraclavicular region
Palmoplantar hyperpigmentation^7^	Common and more frequent with greater skin pigmentation		Small, well-defined hyperchromic macules, variable in number and size, on the palmoplantar surfaces
Melanonychia striata^7^, ^24^	More frequent with greater skin pigmentation; up to 77% of young African-American adults and nearly 100% of those over 50 years, increasing in intensity with age	Melanocytic activation in the matrix, with melanin deposition on the nail plate. Less commonly, melanocytic hyperplasia in the nail matrix or bed	Pigmentation in longitudinal or diffuse bands on the nail, from the matrix to the tip, more common in the 1^st^ and 2^nd^ fingers. On dermoscopy, homogeneous grayish background, with longitudinal parallel grayish lines. Pseudo-micro-Hutchinson sign (cuticle pigmentation) may be seen
Hyperpigmentation of the oral mucosa^7^	Develops during the first 2 decades of life	Melanocytic activation with melanin deposition in the epithelium	Hyperchromic patches, localized or disseminated. In the gums, they are more common and unaesthetic, especially when there is a high smile line
Mongolian spot^25^	40% to 90% of black newborns. Usually disappears by the age of 7	Unknown. When extensive, it is associated with systemic conditions, such as congenital errors of metabolism	Blue-gray spot, usually single, on the lumbosacral region. Melanocytes in the dermis.
Oral leukoedema^26^	70% to 90% of black adults and 50% of children; more frequent in men	Physiological, but irritation caused by smoking or poor oral hygiene are factors. There is accumulation of fluid in the epithelial cells	Grayish-white, edematous plaques on the buccal mucosa, seen bilaterally, which disappear if the mucosa is distended. White folds or lines may cross the affected area
Pearly penile papules^27^	14% to 48% of black men; in post-pubertal age or early adulthood, diminishing with age	They are believed to be vestigial structures	Asymptomatic, benign, pearly-white, dome-shaped or filiform papules, measuring 1 to 4 mm, around the glans, in single or double row. Dermoscopy shows a cobblestone or bunch of grapes pattern with dotted or comma-shaped central vessels. Histopathology shows it to be angiofibroma

## Dermatoses with higher incidence on black skin

Five major dermatology diagnoses stand out in African-Americans: acne, unspecified eczema, atopic dermatitis, seborrheic dermatitis, and dyschromia.^28^

In the Angolan population with phototype V‒VI, in Luanda, the prevalence of acne, dermatophytosis and atopic dermatitis stands out. In individuals aged 13 or over, the main complaints were acne (23.6%), dermatophytosis (11.0%) and pityriasis versicolor (8.6%). In the pediatric population, atopic dermatitis (29.4%), tinea capitis (13.7%) and molluscum contagiosum (12.5%) were the most common disorders. In adult women, acne (31.3%) was the main condition, while in adult men, it was dermatophytosis (13.5%).^29^

Sociocultural habits in black communities, aiming to mask changes in pigmentation with emollient products, produce folliculitis. The use of ointments, oils and creams on hair generally causes alopecia and cosmetic acne in the frontal region.^7^

### Acne

Acne is one of the most common conditions seen in black children and adolescents. Its pathogenesis is similar among different ethnic groups, but products frequently used on the skin and hair by black individuals may be comedogenic. Nodulocystic lesions are less frequent, but even lesions without clinical inflammation, such as comedones, have a high degree of inflammation histopathology, which may explain post-inflammatory hyperpigmentation. Keloid scars are frequent sequelae on the chest, back and jawline.^1^, ^30^, ^31^

### Eczema

#### Contact eczema

Black individuals are less susceptible to irritants. This is attributed to the greater cohesion of the stratum corneum cells in dark skin. This difference is undetectable if the stratum corneum is removed.^7^ The clinical characteristics in patients with dark skin include lichenification, hyperpigmentation, and early papular or follicular eruption with minimal erythema.

In patch tests, there is a similarity in the general frequency of allergies between black and white individuals, but there are differences regarding sensitization to allergens, with positivity for nickel, fragrance mix, bacitracin, balsam of Peru and paraphenylenediamine (PPD). PPD, found in hair dyes, and imidazolidinyl urea, a formaldehyde-releasing preservative, were more frequently allergenic in black men compared to white men.^32^ The use of darker hair dyes in the black community may explain the greater sensitization to PPD and differences in sensitization patterns are likely due to cultural practices.^33^

### Atopic dermatitis (AD)

The pathogenesis of AD includes genetic and environmental factors, varying among different ethnic and racial populations. Black individuals are seven times more likely to seek care for AD than white ones.^34^ There are differences in the skin barrier properties that may have implications for AD.^28^ Differences in *S. aureus* strains, including variability in the presence of superantigen genes, have been demonstrated between whites and blacks with AD.^35^ Clinically, there are different expressions of erythema, due to background melanin, manifesting as reddish-brown, violet, gray or intensely hyperchromic plaques, instead of bright red. There is greater visibility of desquamation and dry skin. They are more frequently located in the extensor areas than in the flexural areas and pruritus is more frequent and more severe. Follicular and perifollicular papules, lichenoid or nodular-papular morphologies are common, in addition to periorbital lichenification and hyperchromia. Post-inflammatory hyperpigmentation is more frequent and persistent.^35^

### Seborrheic dermatitis (SD)

SD is among the five most common diagnoses in black people.^29^ In adults and adolescents, the underlying erythema is barely visible and there is hypopigmentation in the classic areas of involvement,^36^ possibly due to the inhibition of tyrosinase function in melanocytes by *Malassezia* metabolites, with less pigment production.^37^, ^38^ Black children often present with erythema, desquamation and hypopigmentation of the affected areas and overlying AD, which accentuates the hypopigmentation.^37^, ^39^

Scalp SD is prominent among black women and can be exacerbated by excessive use of hair oils or ointments and infrequent washing.^38^ Children generally do not have the “cradle cap” seen in Caucasians.^38^, ^39^ In other age groups, petaloid SD, with circinate lesions, polycyclic coalescing rings, either pink or hypopigmented, with furfuraceous desquamation, is observed on the hair implantation line or on other areas of the face.^39^

### Dyschromias

Pigmentary disorders are the third most common condition in black skin,^28^ and can be either hyperpigmentation or hypopigmentation/achromia, with the latter being psychologically devastating, due to the marked contrast with normal skin, in addition to resistance to treatment.^7^

#### Post-inflammatory hyperpigmentation (PIH)

**Figure 2:**
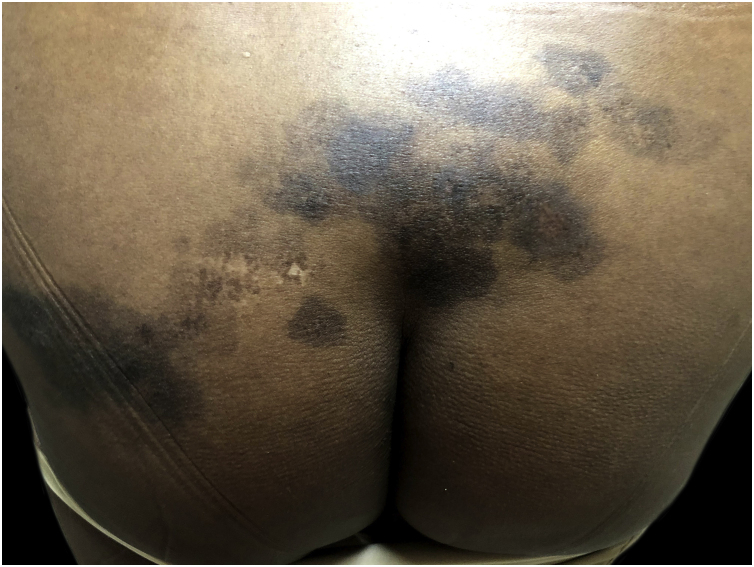
Post-inflammatory hyperpigmentation: hyperchromic macules on the lumbosacral and gluteal regions.

Among pigmentation disorders in black skin, PIH is the most common. It occurs after skin inflammation or injury,^30^, ^40^, ^41^ resulting from excessive production or irregular dispersion of melanin.^2^ There are several etiologies, including infections, inflammatory diseases and skin injury caused by physical or chemical agents, including cosmetic procedures. PIH manifests as macules in the same distribution as the initial inflammatory process, the intensity of which may have a direct correlation with phototypes (Fig. 2).^41^

#### Post-inflammatory hypopigmentation

Post-inflammatory hypopigmentation is more common and more evident in people with dark skin. It results from inflammatory (AD, SD, contact dermatitis, psoriasis, etc.), neoplastic (mycosis fungoides), infectious (syphilis), traumatic (wounds, burns) or iatrogenic conditions (post laser, cryotherapy or chemical peelings). It appears as single or multiple hypochromic macules, without desquamation.^7^

#### Progressive macular hypomelanosis (PMH)

PMH is a common and poorly diagnosed dermatosis. It is distributed worldwide, being more common in tropical countries in black people, preferentially affecting young women. *C. acnes* is believed to produce a depigmenting factor, as this bacteria can be grown from follicles in hypopigmented areas, but not from neighboring normal-appearing skin. It is characterized by confluent, ill-defined, non-desquamative hypopigmented patches located on the trunk, around the midline and, rarely, extending to the proximal extremities and head and neck regions.^31^ There is no pruritus, pain or previous inflammation; it is stable or progresses slowly for years, with spontaneous disappearance later in life.^31^ Wood's lamp examination may show red follicular fluorescence in hypopigmented areas. Dermoscopy reveals whitish, poorly defined areas without desquamation.^42^ Histopathology shows a decrease in melanin in the epidermis, while the dermis appears normal.^43^, ^44^

#### Pityriasis alba (PA)

PA begins around three to 16 years of age, without sex predilection, occurring more commonly in darker skin.^31^ Its etiopathogenesis remains unknown; however, it is believed to represent nonspecific dermatitis with residual post-inflammatory hypopigmentation. Associated findings include atrophic sebaceous glands, iron-deficiency anemia, and low serum copper levels.^44^ Black children may be genetically predisposed to xerosis, which can lead to PA.^31^ Environmental factors such as sun exposure, long and frequent baths and mechanical exfoliation seem to be predisposing factors on susceptible skin.^31^ It is important to inquire about personal or family history of atopy, as it is one of the minor criteria for AD.^31^, ^44^ In dark skin, early lesions present as subclinical dermatitis and erythema is barely evident.^31^ The initial lesions are desquamative papules that evolve into clearly visible hypochromic areas, different from lightly pigmented skin, due to the contrast with healthy skin. It generally affects the face, neck and arms; histopathological findings are nonspecific.^31^, ^44^

#### Melasma

Melasma is a common acquired hyperpigmentation, typically occurring on the face, with a high prevalence in women and darker skin types. Family history, especially in darker skin, is an important risk factor.^45^, ^46^ Clinically, the pigmentation is more intense and persistent. On histopathology, melanophages have been detected in the superficial dermis, with a very heterogeneous distribution, varying from one lesion to another in the same patient and even within the same area of melasma.^46^

## Dermatoses highly specific for black skin

### Pseudofolliculitis barbae (PFB)

PFB, also known as pili incarnati or sycosis barbae, is a common chronic disease, seen almost exclusively in black men, between 14 and 25 years of age, with a prevalence between 45% and 83%. It causes problems in professions that require a clean-shaven appearance.^47^ It is a foreign body type inflammatory reaction around the hair due to its structural characteristics and growth orientation, which curves downwards and penetrates the epidermis and dermis (extrafollicular penetration) or grows inside the follicle, perforating its wall and penetrating the dermis (transfollicular penetration). The latter generally results from an incorrect shaving technique, such as stretching the skin, and the use of blades that pull the hair instead of cutting it, causing retraction with the tip below the skin surface. Molecular biology studies detected the substitution of a single nucleotide in the specific keratin of the hair follicle layers (K6hf).^48^ The mutation in K6hf is believed to lead to a weakened cytoskeleton, increasing the chances of ingrown hairs. Carriers of the A12T polymorphism of the gene that encodes keratin have a six-fold greater chance of developing PFB. About 76% of men who shave regularly and who carry the A12T polymorphism develop PFB, making this a significant risk factor for the disease. Finally, PFB has been associated with the use of cyclosporine and corticosteroids in kidney transplant patients and also with violin pressure under the jaw (violinist's neck).^49^ Clinically, they are follicular papulo-pustules, where the hair can be seen in a linear groove. It mainly affects the face and neck of men, but it is also seen in women in areas where hair removal is practiced, such as the armpits, pubic region and legs during menopause or in hirsute young women. Pustules may be secondary to infection, usually by *Staphylococcus epidermidis*. Post-inflammatory hyperpigmentation and keloids may occur. Histopathology shows invagination of the epidermis, neutrophilic inflammatory infiltrate, microabscesses, foreign body-type granulomas and fibrosis.^49^

## Keloid

Keloids and hypertrophic scars are considered distinct types of scars. They are benign excessive growths of scar tissue at sites of skin injuries. Clinically, keloids are smooth, shiny and firm, red or pink papules, plaques, or nodules, with progressive hyperpigmentation, that spread into the surrounding normal skin. Hypertrophic scars do not grow beyond the limits of the original injury. On histopathology, keloids tend to have a large volume of thick, eosinophilic, hyaline collagen and many blood vessels, the opposite occurring in hypertrophic scars. However, the scar may present the growth pattern and histopathological characteristics of both hypertrophic scars and keloids, suggesting that the two types of scars are manifestations of the same fibroproliferative disease, differing only in the intensity and duration of inflammation.^50^, ^51^

### Dermatosis papulosa nigra

**Figure 3:**
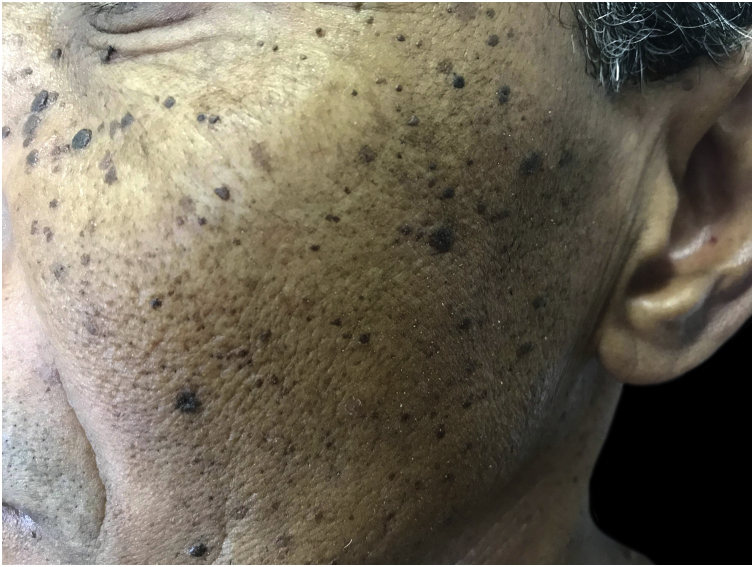
Dermatosis papulosa nigra: hyperchromic papules on the face.

These are benign skin lesions of unknown etiology, but with a genetic predisposition, with more than 50% of patients reporting a family history.^7^ The highest prevalence is found in black people, between 10% and 30%. It begins after puberty, as multiple hyperpigmented, rounded or filiform papules, measuring 1 to 5 mm, on the face, neck and trunk, gradually increasing in number (Fig. 3). They are generally asymptomatic, although they can cause skin irritation and pruritus.^7^

### Sickle cell anemia ulcers

Ulcers are a common complication of sickle-cell disease. Geographic origin is a factor in the occurrence of this disease.^52^, ^53^ The most common genotypes are, in decreasing order of frequency: homozygous SS sickle cell anemia, compound heterozygous Sb0 thalassemia, and sickle cell trait hemoglobin. The incidence of ulcers varies with the total hemoglobin level and the fetal hemoglobin level. When low, they are associated with an increased risk. These are very painful ulcers, located on the legs, which are difficult to heal. The prognosis is associated with its clinical characteristics, and not with the biological characteristics of sickle cell disease. Complete healing was achieved within six months in 75% of cases, when the area was less than 8 cm^2^ and the duration was less than nine weeks, comparable to the healing rates observed in venous ulcers.^52^, ^53^

### Ainhum

Also known as dactylolysis spontanea or ayun (in the Yoruba language, it means ‘cutting’), it occurs worldwide, but is more common in African countries, with a low prevalence (0.015%). In Brazil, it affects individuals with different phenotypes.^54^

It begins with an asymptomatic fissure at the base of the fifth toe, evolving into a fibrous constrictive painful ring leading to mutilation due to ischemia. Occasionally, it may be bilateral.^55^, ^56^ It differs from pseudo-ainhum, secondary to conditions that cause constriction, by its spontaneous and idiopathic onset. The clinical diagnosis includes at least one of the following three criteria: soft tissue constriction, bulbous enlargement of the toes, and thinning or lysis of the phalanx bones, and requires radiographic confirmation.^56^

### Confluent and reticulated papillomatosis (CRP) of Gougerot-Carteaud

Dermatosis that affects adolescents and young adults, especially women, twice as common in black people than in whites. It may be associated with insulin resistance and obesity.^31^ It is characterized by thin, scaly papules, initially erythematous and later brown, which merge into reticulated, hyperkeratotic or verrucous plaques on the chest and back, asymptomatic or slightly pruritic. Histopathology shows hyperkeratosis, with irregular papillomatosis, focal acanthosis and increased melanin. Acanthosis nigricans is the main differential diagnosis.^31^

### Hair and scalp diseases

#### Fragile and brittle hair

Breakage is a common problem in black hair, leading to deficient growth and alopecia, due to the intrinsic characteristics of fragility and reduced tensile strength. Fragility results from the elliptical shape of the shafts originating from the curved follicle, different hair diameters and dry scalp. Transverse asymmetry, with points of weakness, reduces tensile strength.^57^, ^58^ Damage to the hair shaft resulting from the removal of proteins or 18-methyl eicosanoic acid (18-MEA), the main lipid of the hair cuticle, contributes to breakage. of hair, secondary to excessive use of heat or chemical agents, in addition to hairstyles that exert traction.^17^, ^59^, ^60^

Dermoscopy (trichoscopy) shows trichorrhexis nodosa, where the shaft is nodularly dilated and lighter, and distal trichoptilosis (cracking of the ends) or central trichoptilosis (longitudinal division in the central part of the hair shaft).^17^, ^61^, ^62^, ^63^

#### Traction alopecia

Caused by repetitive tension in hair from braids, ponytails, turbans, buns, dreadlocks, weaves, extensions, among others,^64^ traction alopecia generally begins in childhood.^64^, ^65^ The pattern is characteristic, reflecting the distribution of traction, affecting the temporal, pre-auricular and above-the-ears regions, and may involve other parts of the scalp. Folliculitis, reduced hair density and broken hairs are seen in these areas, in addition to the fringe sign (long terminal hairs anterior to the alopecia area, which consist of fine or miniaturized residual hairs). It may be associated with headache, relieved by loosening the hair.^65^, ^66^ Early diagnosis is important, when it is still reversible. Trichoscopy in the early stage shows the honeycomb pattern of the preserved scalp, perifollicular erythema, reduced hair density and vellus hairs. The advanced stage is characterized by loss of follicular openings.^17^, ^65^, ^66^ Histopathology in the initial stage shows a normal number of hair follicles, but a greater number of telogen and catagen follicles, in addition to trichomalacia (remnants of hair shafts in the ostia). In advanced stages, there is a decrease in the number of terminal follicles, replaced by fibrous tracts. There is no inflammatory infiltrate during the course of this disease.^66^

#### Folliculitis keloidalis nuchae

**Figure 4:**
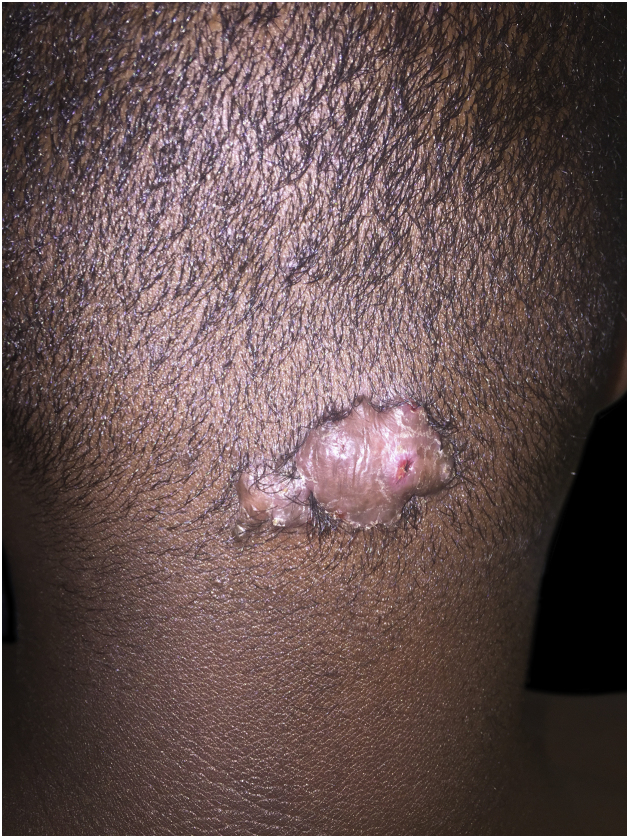
Keloid folliculitis of the neck: sessile nodule on the back of the neck.

It results from ingrown hairs, extrafollicular or transfollicular penetration of the skin, generally secondary to the cutting of black hair.^67^, ^68^ It presents as papules, pustules, plaques and nodules on the posterior region of the scalp, commonly associated with alopecia and keloids. Trichoscopy shows broken hairs with tufts, ingrown hairs and peripilar casts (Fig. 4).^67^, ^68^

#### Dissecting folliculitis (cellulitis)

Also called folliculitis and perifolliculitis *capitis abscedens et suffodiens*, it is a chronic scalp disorder, observed mainly in young men. The lesions, initially papulopustular, develop into painful, multifocal inflammatory nodules, connected by intercommunicating sinuses, resulting in cicatricial alopecia.^7^, ^69^ On trichoscopy, characteristics of non-scarring alopecia are initially observed, with regularly distributed pinpoint white dots, enlarged follicular openings, “3D” yellow spots (accumulation of sebum and keratin), black spots and fractured hair shafts. In the terminal stage, cicatricial lesions appear, characterized by ivory-white confluent areas, without follicular ostia.^68^, ^69^, ^70^

#### Central centrifugal cicatricial alopecia (CCCA)

Formerly known as hot comb alopecia (a method of straightening hair), pseudopelade, and follicular degeneration syndrome, CCCA is most common in young to middle-aged women of African descent. The etiology is unknown but with an apparent influence of genetic factors, traction, heat and chemicals. It is characterized by a chronic and progressive lesion on the vertex of the scalp, which progresses in a centrifugal and symmetrical manner, with activity in the peripheral zone, surrounding the central zone of cicatricial alopecia.^7^, ^71^, ^72^, ^73^ Signs of inflammation are absent, although erythema, follicular pustules and desquamation may appear. It is usually asymptomatic, but pain, pruritus or burning sensation in the scalp may occur.^73^, ^74^ There is a spontaneous reduction in inflammation after years or decades.^75^ Trichoscopy reveals a preserved pigmented honeycomb network and irregularly distributed white dots. A grayish-white peripilar halo is a specific and sensitive sign, corresponding on histopathology to follicular ostia surrounded by fibrosis.^74^, ^75^ Other characteristics include hair shaft variability, perifollicular erythema, broken hairs as black dots within the follicular openings or as short broken shafts, asterisk-like pigmented macules with sparse, vellus-like terminal hair shafts and scattered white patches.^74^, ^75^, ^76^ In the early stage histopathology reveals a perifollicular lymphocytic infiltrate with perifollicular fibroplasia. In later stages, there is destruction of the follicular epithelium and fragments of hair shafts sorrounded by granulomatous inflammation, followed by replacement of the follicles by connective tissue besides tufting/polytrichia of the hair follicles.^76^

## Some dermatoses with peculiar aspects in black skin

### Sarcoidosis

African Americans tend to develop sarcoidosis at a younger age and are at greater risk of developing severe lung and skin disease. Lupus pernio is associated with a worse prognosis for both systemic disease and more chronic skin lesions.^77^, ^78^ Black people have sarcoidosis at a younger age, on average around the 40 s, and women more so than men. Most of the time, the initial lesions are asymptomatic, with the first consultation being motivated by disfigurement. Papules on the face and neck are the main observed lesions. Erythema nodosum is less common than in white individuals. There is a greater propensity for more severe disease, with a greater number of extracutaneous organs being involved, in the following order: lungs, lymph nodes and heart. Mortality rates are higher.^77^, ^78^ Lupus pernio is associated with a worse prognosis for both systemic disease and more chronic skin lesions.^7^

### Lichen planus (LP)

Hypertrophic, pigmentosus, and actinic types of LP are characteristic of dark-skinned individuals.^7^ The violaceous color, typical of light skin, is less visible on dark skin and PIH is intense. On dermoscopy, the most common features include: pigmentary changes, pearly whitish structures and a purple background.^79^

#### LP pigmentosus (LPP)

**Figure 5:**
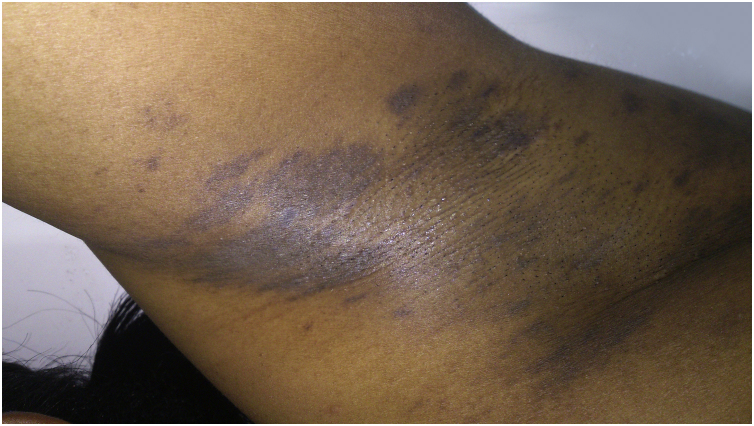
Lichen planus pigmentosus: slightly raised papules and bright hyperchromic macules in the axilla.

LPP is a rare variant of LP, which begins after the age of 30, predominantly in black women.^80^ Although the pathogenesis is the same as in classic LP,^81^ sunlight is an important factor, in addition to chemical agents (contactants and drugs) and microorganisms.^80^, ^81^ The initial lesions are small round or oval macules, gray or brown, with irregular ill-defined edges, which coalesce to form diffuse, reticulated, perifollicular or annular pigmented areas,^80^, ^82^ distributed mainly in body regions exposed to the sun, starting on the face and neck, spreading to the upper extremities and trunk, symmetrically, rarely affecting the oral mucosa, the scalp, nails, palms and soles.^80^, ^82^ There are no Wickham striae.^82^ Eventually, there are classic LP lesions. Pruritus is mild or absent.^81^ One variant is LPP inversus, which affects skin not exposed to the sun, particularly flexural and intertriginous areas, in which a transition from papules to macules can be observed (Fig. 5). Mechanical stimuli are triggers. Other variants are: localized, segmental, linear, zosteriform and oral mucosa LPP.^80^, ^81^, ^82^

Dermoscopy of LPP inversus shows homogeneous brown areas (epidermal pigmentation), as well as brown-gray or blue-gray dots and globules (pigmentary incontinence), which are initially grouped into a diffuse pattern of black pigmentation. Over time, they converge forming reticular, linear and cobblestone patterns. White dots are secondary to lack of pigmentation in the follicular openings, and the absence of pigment in the grooves may be due to lack of exposure to friction.^82^

Histopathologically, the lichenoid inflammatory infiltrate is similar to the one seen in classic LP,^81^ but it regresses quickly, without the proliferation of keratinocytes, and the melanin deposited in the dermis remains for months to years.^82^ Direct immunofluorescence is rarely positive, with IgM and C3 at the dermal-epidermal junction and IgG and IgA in keratinocytes.^81^, ^82^ It can be associated with hepatitis C, endocrinopathies, autoimmune diseases and malignancies, as well as other variants of LP.^80^, ^81^, ^82^

### Psoriasis

**Figure 6:**
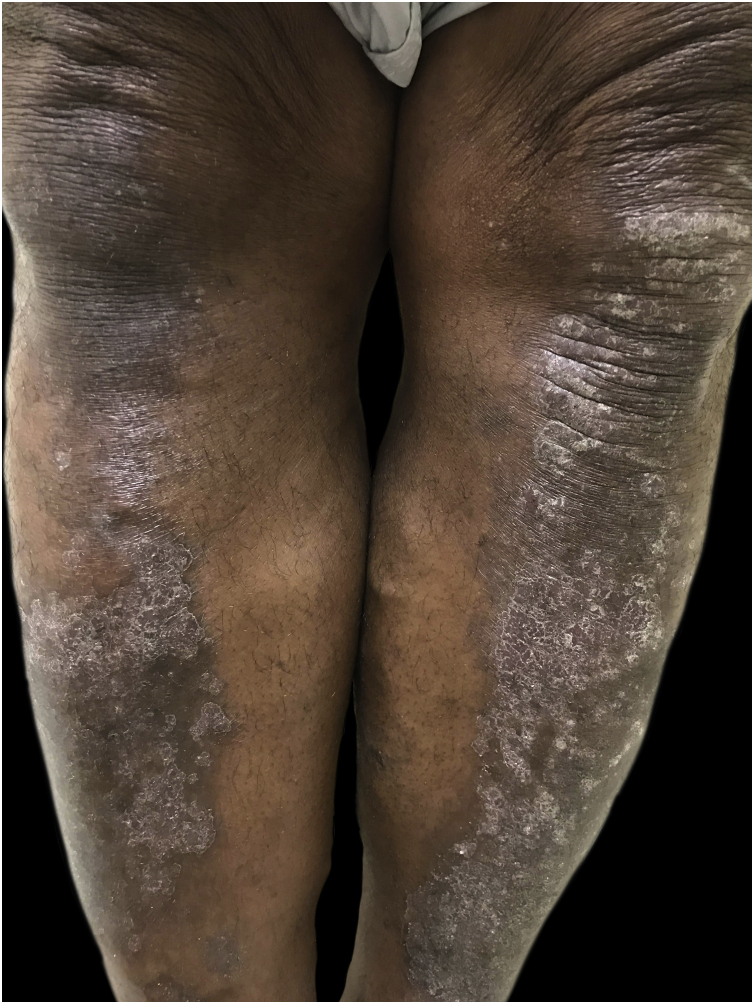
**Psoriasis:** hyperchromic plaques with thick symmetrical scales on the anterior surface of the legs.

Psoriasis in dark-skinned patients is characterized by less distinguishable erythema, thicker plaques, greater desquamation and greater body involvement (Fig. 6). The resolution of the lesions results in pigmentary changes (hyper- and hypopigmentation). The frequency of psoriatic arthritis in blacks is approximately half that in Caucasians.^83^ The most common dermoscopic features include: light red background, red dotted vessels, regular vessels, white scales, irregular distribution, and pigmentary changes.^79^

### Lupus erythematosus (LE)

**Figure 7:**
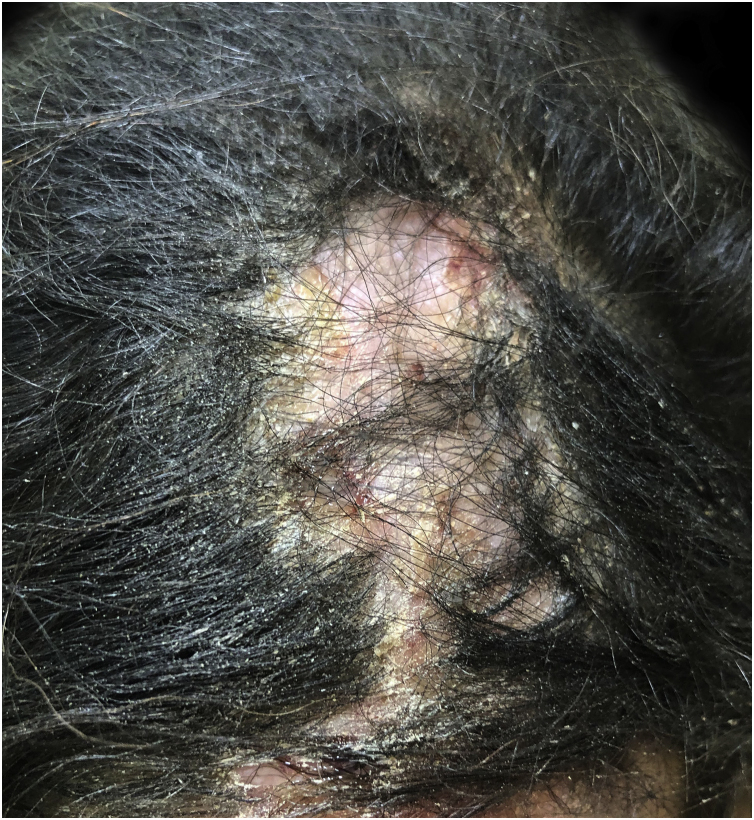
Discoid lupus erythematosus: atrophic alopecic plaque on the frontoparietal region.

Discoid LE (DLE) has a higher incidence and severity in black women.^84^ The lesions are mostly located in exposed areas, with greater involvement of the scalp and ears, with depigmentation and cicatricial alopecia being prominent (Fig. 7).^84^ Lesions vary from desquamative to hypertrophic or verrucous and tend to have an annular morphology. Depigmentation is obvious and can be disfiguring.^84^ PIH is also prominent,^84^, ^85^ with a risk of hypertrophic scars. Hair care practices contribute to more frequent and severe cicatricial alopecia.^84^

The prevalence of systemic LE (SLE) is approximately three-fold higher among black people, with women being most affected.^86^ The disease shows rapid progression and greater severity, with kidney and cardiovascular damage and higher and earlier mortality rates.^86^, ^87^ The causes of these disparities are multifactorial, including genetic, epigenetic, socioeconomic, and racial discrimination ones.^87^ Cutaneous involvement occurs in around 80% of cases, the majority due to specific lesions, which are associated with less severe disease.^88^ Among non-specific lesions, Raynaud's phenomenon, oral ulcers and diffuse melanonychia affect almost 40% of the patients. Acute lupus erythematosus, the most common form of SLE in Caucasians, is uncommon in black individuals, and is associated with kidney disease, livedo reticularis, non-cicatricial alopecia, and oral ulcers. DLE occurs in about 50% of cases and is associated with arthritis. Subacute lupus erythematosus is rare, perhaps because subtle erythematous eruptions and photosensitivity are difficult to detect.^89^

### Vitiligo

**Figure 8:**
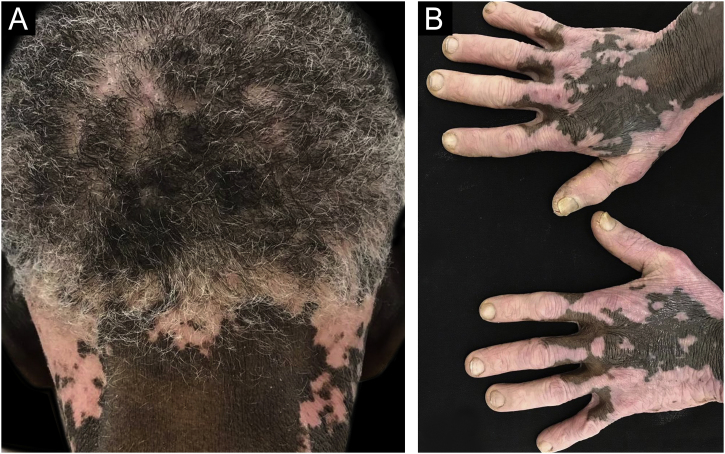
Vitiligo: achromic macules on the scalp and neck (A) and on the dorsum of the hands (B).

Vitiligo is very debilitating in dark skin due to its highly inaesthetic characteristic, with a profound effect on the patients quality of life. Its prevalence varies greatly within the same race and among different races. In addition to the usual etiopathogenic factors, Koebner phenomenon is implicated in dark skin. The lesion occur mainly in areas exposed to the sun, especially the lips. The genitals are also often affected. Depending on the intensity of the acromia, speckled and trichromatic vitiligo seems to be specific for black skin, with a high contrast between lesions and healthy skin (Fig. 8).^90^

### Syphilis

**Figure 9:**
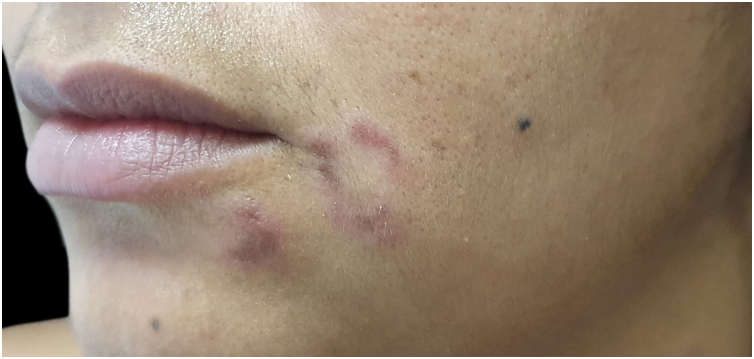
Elegant syphilis: erythematous papule and annular plaque on the face.

Secondary syphilis in black people sometimes shows intense desquamation, giving the lesions a psoriasiform appearance. On the face, the papules tend to cluster around the nose and mouth, simulating seborrheic dermatitis. Periorificial lesions assume characteristic annular and circinate configurations (elegant syphilis; Fig. 9). In the inguinocrural region, due to friction and humidity, the papules, rich in treponemas, become vegetative and macerated (flat condyloma). On the oral mucosa, white vegetative plaques can be observed on an eroded base, and are contagious.^91^

**Figure 10:**
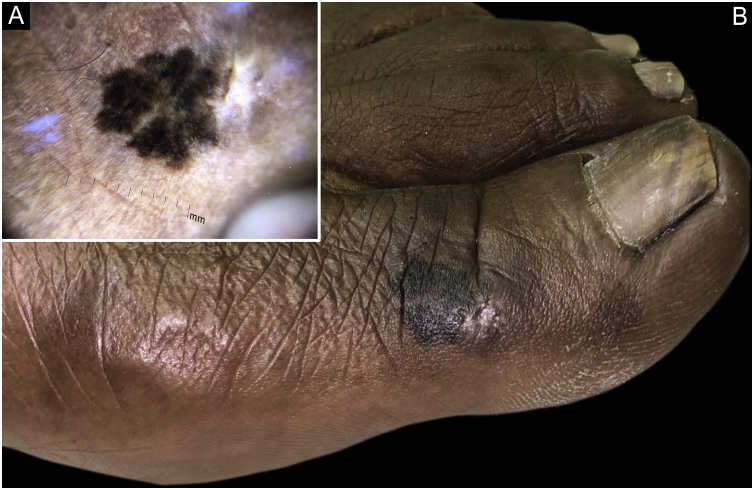
Acral melanoma: blackish macule on the hallux (B). Dermoscopic appearance (A).

### Pityriasis versicolor (PV)

Some studies report the predilection of PV for individuals with darker skin. It initially presents as round or oval hypopigmented or, less frequently, hyperpigmented macules, measuring 3 to 5 mm, symmetrically distributed on the upper trunk, shoulders or flexural areas. Facial and cervical involvement is more common. Subsequently, they spread and coalesce to form larger, irregularly shaped patches. When active, there is subtle desquamation associated with pruritus. After treatment, pigmentary changes without desquamation may persist for months.^92^

#### Neoplasms

Although the risk for skin cancer is lower in black people, exposure to UV rays is also a risk factor.^30^ The repair of DNA damage induced by UV radiation is more efficient in black people. ^93^ Black individuals are less likely to use sun protection, as they believe it is unnecessary.^30^ Table 3^93^, ^94^, ^95^ illustrates the main characteristics and differences between non-melanoma and melanoma cancer in black skin.

**Table 3:** Main characteristics of non-melanoma and melanoma skin cancers.^30^, ^93^, ^94^, ^95^

Skin cancer	Incidence in blacks	Clinical presentation	Risk factors
BCC	Around 80% of skin cancers.	Solitary pearly papule or nodule, with telangiectasias that are difficult to observe.	UV radiation, albinism, ulcers, exposure to ionizing radiation, ingestion of arsenic, oral psoralen, immunosuppression, among others.
	Second most common.		
	Predominance in females.	Pigmented variants are more prevalent	
SCC	Around 20% of skin cancers.	Erythematous, hyperkeratotic papules, plaques or nodules, generally in places not exposed to the sun (scalp, lower extremities, anogenital area).	Chronic healing processes such as burn scars and chronic ulcers, HPV (including subtypes 16 and 18), immunosuppression, radiotherapy, albinism, epidermodysplasia verruciformis.
	The most common		
Melanoma	The least common	Hyperpigmented macule, plaque or nodule.	UV radiation.
	Lower incidence rate in women (0.9/100,000/year).	Acral lentiginous (palms, soles, nails) is the most common (Fig. 10).	
		Late diagnosis.	

BCC, Basal cell carcinoma; SCC, Squamous cell carcinoma; HIV, Human immunodeficiency virus; HPV, Human papilloma virus; UV, Ultraviolet.

#### Primary cutaneous T-cell lymphoma (CTCL)

Mycosis fungoides (MF) is the most common type of lymphoma in black skin, accounting for more than 50% of CTCLs,^30^ with an incidence rate of 4.0 and 6.1/100,000 for white and black patients, respectively.^96^, ^97^ Black individuals present with MF at an earlier age and at a higher T stage than white ones. Clinically, MF generally presents as patches, plaques or tumors located, preferably, in covered areas, but can also present with erythroderma. The lesions are hyperpigmented in darker skin, rather than erythematous, with a hypopigmented variant. Hyperpigmented lesions correlate with shorter survival, while hypopigmented ones correlate with longer survival and an indolent course.^96^

Sezary Syndrome (SS) is a rare leukemic variant of CTCL, defined by the triad of erythroderma, neoplastic T cells (Sezary cells) in the blood, and lymphadenopathy. Like MF, SS also has a higher prevalence in black individuals compared to white ones. As on lightly pigmented skin, it can be difficult to differentiate it from other diffuse eruptions. The biggest concern is that pruritus and changes secondary to scratching are attributed to xerosis.^96^, ^97^

#### Endemic Kaposi sarcoma

It is limited to sub-Saharan Africa and is typically seen in young, HIV-negative black men, aged 25 to 40. Clinical manifestations can range from indolent to aggressive and lethal. Its presentation can be locally invasive or aggressive with/or without mucocutaneous involvement. There are four subtypes: benign nodular; locally aggressive with invasion of soft tissues and bones; florid and disseminated with the involvement of skin and viscera; and lymphadenopathic, spreading rapidly to lymph nodes and viscera. The last is the most aggressive and is associated with African children aged one to five years.^7^, ^98^

#### Dermatofibrosarcoma protuberans (DFSP)

DFSP is a rare, slow-growing, spindle cell neoplasm located in the dermis or subcutaneous tissue. The incidence among black people is twice as high and the tumors are larger than in white individuals, one explanation being the delay in diagnosis.^99^ It is more common in the trunk than in the head and neck, with no difference for the extremities.^100^ The risk of death in black individuals is 1.7-fold greater than in whites and depends on size.^99^, ^100^

There is a rare, pigmented variant, called Bednar tumor, which occurs in 5% of cases, mainly in blacks,^100^ observed in young and middle-aged adults, occasionally in the pediatric age group. Histogenesis is unexplained and may be related to remnants of embryonic breast tissue or local trauma, with two theories having been proposed: neuroectodermal differentiation or melanocytic colonization.^99^, ^100^ It has been reported in association with dermal melanocytosis (nevus of Ito) and is believed that it originates from a neuromesenchymal cell. It is most commonly located on the trunk, especially on the shoulders and back, and can also affect the head and neck, as well as the extremities. 99, 100 Clinically, it resembles a keloid. The majority appear as masses, plaques, or protruding, firm, multilobulated nodules, with a wide base and slow growth, in the dermis and subcutaneous tissue. On histopathology, it consists of spindle cells, with mild pleomorphism.^99^, ^100^ Pigment-laden dendritic cells differentiate this lesion from conventional DFSP.^100^ Immunohistochemistry detects CD34 positivity in the cells.^99^ The biological behavior is that of intermediate malignancy and it must be differentiated from other pigmented, fusiform skin lesions.^99^, ^100^

## Financial support

None declared.

## Conflicts of interest

None declared.

## Authors' contributions

Maurício Mota de Avelar Alchorne: Design and planning of the study; critical review of important intellectual content; effective participation in research orientation; approval of the final version of the manuscript.

Katleen da Cruz Conceição: Critical review of the literature; data collection; drafting and editing of the manuscript; approval of the final version of the manuscript.

Leonardo Lora Barraza: Critical review of the literature; collection of data; drafting and editing of the manuscript; approval of the final version of the manuscript.

Marilda Aparecida Milanez Morgado de Abreu: Design and planning of the study; critical review of important intellectual content; drafting and editing of the manuscript; approval of the final version of the manuscript.
